# Ruptured Teratoma and Chemical Peritonitis

**DOI:** 10.5334/jbsr.1677

**Published:** 2019-01-17

**Authors:** M. El moussaoui, L. Médart, F. Goffin

**Affiliations:** 1 University of Liège, BE; 2 Department of Radiology, CHR Liège, Liège, BE; 3 Department of Obstetrics and Gynecology, CHR Liège, Liège, BE

**Keywords:** Chemical peritonitis, Mature ovarian teratoma

## Case Report

A 31-year-old nulliparous woman presented to the emergency room with a one-month history of relapsing diffuse abdominal pain and bloating. She was non-febrile, and vital signs were stable. Abdominal examination revealed distension and diffuse tenderness. Blood analysis on admission showed increased total white blood cell count (11.470/mm^3^) and C-reactive protein (39.9 mg/l).

**Figure 1:**
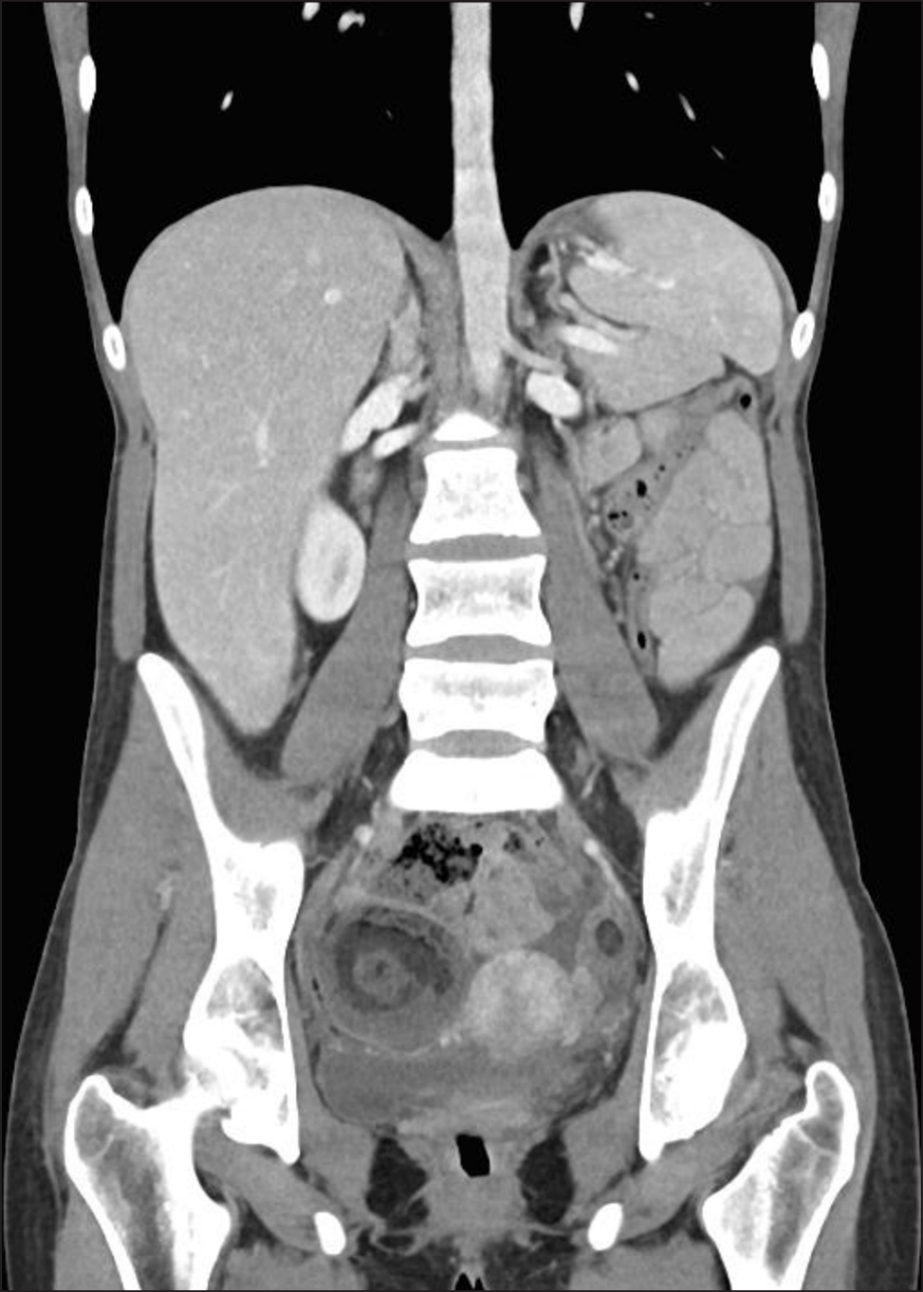


**Figure 2:**
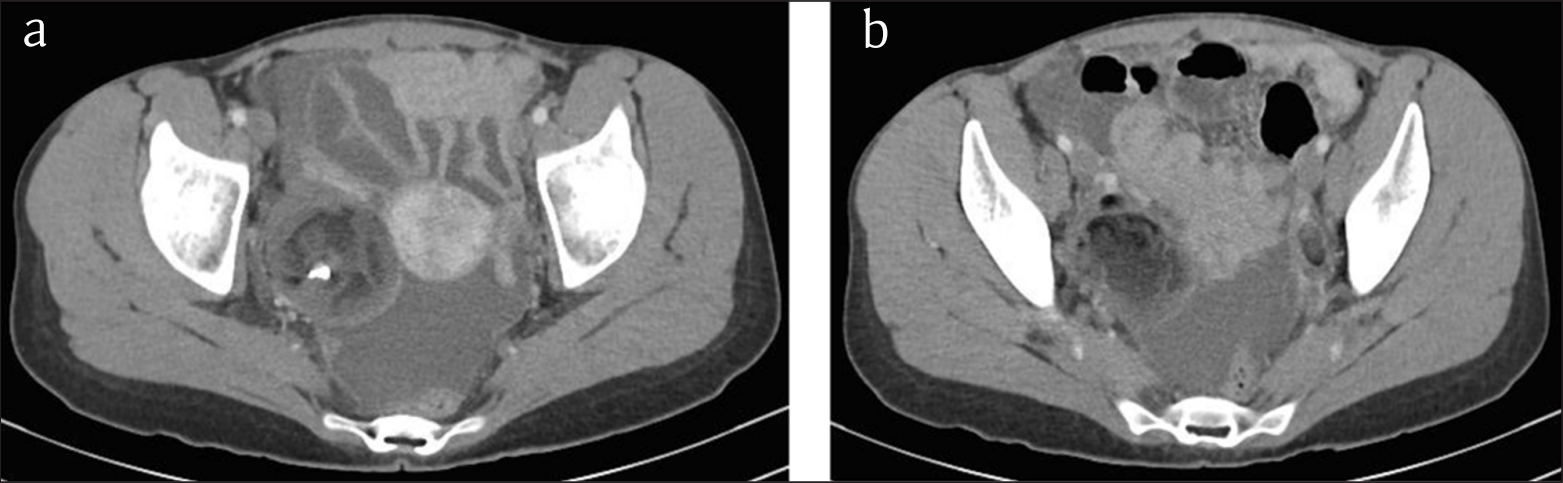


Abdominal computed tomography (CT) demonstrated a heterogeneous right adnexal mass measuring 57 × 53 mm with fatty components and calcification (Figure 1a, arrow), consistent with a mature cystic teratoma of the ovary. Rupture of the teratoma was suspected because of a bulging fatty nodule on the anterior side of the lesion and ascites underlining thickened and enhancing peritoneal layers (Figure 1b, arrows). A similar 2 cm left adnexal mass was observed (Figures 1b and 2). These findings are suggestive of bilateral ovarian teratomas with right rupture and chemical peritonitis.

Accordingly, the patient underwent laparoscopic surgery the day after admission, which confirmed the radiological findings. Right ovarian cystectomy and peritoneal lavage were performed. The patient recovered well.

## Comment

Mature ovarian teratoma, commonly called dermoid cyst, is the most frequent benign tumor in premenopausal woman. It is a germinal tumor composed of well-differenciated derivations from at least two of the three germ cell layers. Ectodermal tissue is invariably present. Ovarian teratomas are bilateral in 8% to 15% of cases.

This condition is usually asymptomatic but complications such as adnexal torsion, rupture, malignant transformation, or infection may occur. Spontaneous rupture is a rare complication, with an estimated incidence of 0.3 to 2.5%. Discharge of the sebaceous content into the abdominal cavity irritates the peritoneum and may lead to aseptic peritonitis, occurring in less than 1% of the cases [1].

Mature ovarian teratomas have a wide spectrum of radiological presentation. The typical radiologic finding is intratumoral fat (in more than 90% of the cases). The most common ultrasound finding is a cystic mass with a densely echogenic protuberance (known as Rokitansky nodule) projecting into the cystic lumen; this nodule often contains tooth, hair, bony structures or sebaceous components. Some sonographic signs, such as tip-of-the-iceberg sign (only a part of the lesion is visible due to posterior acoustic shadowing) and dot-dash sign (multiple thin echogenic bands due to hair), help for the diagnosis. Computed tomography (CT) and magnetic resonance imaging (MRI) have excellent sensitivity and specificity, and CT is particularly helpful in bringing out tiny calcifications.

Ruptured ovarian teratoma can be suggested when demonstrating discontinuity of the cyst wall, when a flattened or distorted tumor shape are noted in the presence of ascites and extratumoral fatty nodules. CT is very sensitive for detection of intraperitoneal fatty nodules, frequently around the liver surface.
